# Patient and Family Participation in Clinical Guidelines Development: The Cystic Fibrosis Foundation Experience

**DOI:** 10.2196/17875

**Published:** 2020-07-13

**Authors:** Sarah E Hempstead, Kelsey Fredkin, Cade Hovater, Edward T Naureckas

**Affiliations:** 1 Cystic Fibrosis Foundation Bethesda, MD United States; 2 Community Advisor to the Cystic Fibrosis Foundation Bethesda, MD United States; 3 Department of Pulmonary Medicine University of Chicago Chicago, IL United States

**Keywords:** cystic fibrosis, guidelines, patient participation, patient engagement

## Abstract

Patient and family participation in guideline development is neither standardized nor uniformly accepted in the guideline development community, despite the 2011 Institute of Medicine’s Guidelines We Can Trust and the Guideline International Network’s GIN-Public Toolkit recommendations. The Cystic Fibrosis Foundation has included patients and/or family members directly in guideline development since 2004. Over time, various strategies for increasing patient and family member participation have been implemented. Surveys of recent patient/family and clinical guidelines committee members have shown that inclusion of individuals with cystic fibrosis and their family members on guidelines committees has provided insight otherwise invisible to clinicians.

## Introduction

Cystic fibrosis (CF) is a rare, genetic, life-shortening disease that impacts approximately 35,000 people in the United States [1]. The small population size has resulted in a paucity of evidence addressing many aspects of CF care, which impacts the development of clinical practice guidelines. Cystic Fibrosis Foundation (CFF)-sponsored guidelines bolster limited evidence with clinical and patient expertise. Since its first guideline was published in the peer-reviewed literature in 1992 [2], the CFF has continued to sponsor guidelines to standardize care and improve outcomes for individuals with CF. Guidelines are developed by committees of experts, including members of the CF multidisciplinary care teams and others who treat people with CF. For over a decade, CFF guidelines committees have also included individuals with CF and/or their family members. As experts in life with the disease, they provide essential information about their perspectives and experiences and can provide insights otherwise invisible to the clinical community.

To ensure that the outcomes of the CFF’s work fit within its chronic care model [3], is rooted in patient-centered needs, and recognizes the importance of community and patient engagement [4-6], the CFF has formalized the process of partnering with and including individuals with CF and their family members in guideline development [7,8]. The first individuals with CF and family members included on guidelines committees not only provided details on their lived experience with CF, but also brought their professional experiences in law, quality improvement, and medical writing to the committee. At this time, the CFF was undergoing a cultural shift towards an increased value in partnership between patients, families, and clinicians. As the culture at CFF continued to move towards partnership, those included on guidelines committees began to represent a broader segment of the CF community. A network of CF community members called Community Voice was developed in 2014 to assure that opportunities to participate are available to a broad spectrum of participants. This group, made up of individuals with CF and family members, is involved in shaping programs and initiatives that impact the broader CF community and has helped the CFF foster meaningful engagement and partnership with those it serves [9]. The development of Community Voice has enabled a more extensive range of patients and family members to participate in, or apply for, opportunities to partner with clinicians on guideline development.

## Patient and Public Involvement in Guideline Development and Implementation

The Institute of Medicine’s (IOM) standards for guideline development, published in 2011, call for the inclusion of patient and public involvement (PPI) [10]. The Guidelines International Network (GIN) toolkit, G-I-N PUBLIC, outlines different methodologies of PPI: Participation, Consultation, and Communication [11]. In 2011, a data synthesis found that of the 71 guideline manuscripts reviewed, 39% included PPI in a guidelines working group, 10% in the literature review, 34% in a consultative capacity, and 13% in a public poll or survey [12]. The best method for including PPI in guideline development and implementation has not been determined [13,14].

Below is a description of how the CFF involves the community in the development and implementation of guidelines and how clinicians and community members perceive the impact of their involvement using GIN’s PPI methodology. The learnings from the CFF presented below could be adapted by other guideline developers to incorporate PPI into their process.

### Participation

A recent study comparing parallel groups, one including patient representatives and the other not, concluded that PPI should be an essential part of trustworthy guideline development [15]. Others have argued that the participation of patients and families in all aspects of guideline development is not essential as long as their voice contributes to the determination of key questions addressed by the guidelines [16]. CFF guideline committees look to patient/family participants to provide insight into the priorities and perspectives of individuals with CF, to determine the topics addressed, and to weigh in on the recommendation statements. The inclusion of an individual with CF and/or a family member also offers insight into the real-world implementation of guideline recommendations from their lived experience with the disease. Including an individual with CF and/or a family member of someone with CF rather than a third-party patient advocate, as the community representative on the committee, highlights aspects of the lived experience of which clinicians and patient advocates may not otherwise be aware. The addition of patient preferences through the inclusion of PPI on the committee can inform the guideline development process [17,18], yield a more patient-centric and evidence-based guideline, and may increase care partnerships and the ability to sustain daily care [19].

While the IOM and GIN recommend PPI, a 2017 study found that just 8% of the 101 guideline developers reviewed require PPI on guideline development committees [20]. A 2008 study found 39% (12 of 31), and a 2012 study found 16.7% (19 of 114) of guideline developers included in the study used the participation strategy of PPI in the guideline development group [21,22]. Since 2004, individuals with CF and/or family members have directly participated in the development of all 28 CFF care guidelines by serving on specific guideline committees. Patient and family committee members work with clinicians to develop PICO (person, intervention, comparison, outcome) questions, are encouraged to participate in the literature review with guidance from other committee members, take part in drafting recommendation statements, and vote alongside other committee members on the final recommendation statements. They are encouraged to share their expertise from living with CF and experience with the guideline topic.

### Consultation

Studies have shown that 33% to 45% of guidelines undergo an external review or public comment period that includes patients or general public commenters [20,21]. Starting with the Infection Prevention and Control Guidelines, published in 2014, the CFF has sought public comment on its draft guidelines, including feedback from individuals with CF and their families (Table 1). Before the initiation of Community Voice, draft guidelines were distributed to patients and family members through CFF multidisciplinary listservs, some of which included members of the patient and family community. After the expansion of Community Voice in 2017, the guidelines public comment period became more accessible to individuals with CF and their families. Public comment periods for new guidelines were consistently shared for international distribution in both the clinical and patient/family communities beginning in 2019. Two joint CFF and European Cystic Fibrosis Society guidelines, published in 2015 and 2016, had previously been shared internationally.

**Table 1:** Patient/Family Public Comment Feedback. Starting with the CFTR Modulator guidelines, published in 2018, the guidelines have been distributed through wider communication channels, including Community Voice. Since than the number of patient/family responses has increased.

Guideline (year published)	Individual with CF Responses	Family Members Responses	Total Individual with CF and Family Members with CF Responses to Public Comment
Infection Prevention and Control (2014)	5	9	14
Eradication of Initial Pseudomonas (2014)	2	1	3
Depression and Anxiety (2015)	14	17	31
Nontuberculous mycobacteria (2016)	2	6	8
Preschool Aged Care (2016)	0	16	16
Enteral Tube Feeding (2016)	4	7	11
Diagnosis (2017)	0	5	5
Colorectal Cancer Screening (2018)	2	3	5
CFTR Modulator (2018)	18	30	48
Lung Transplant Referral (2019)	23	11	34
Advanced CF Lung Disease (2020)	19	10	29
Models Palliative Care (TBD)	17	19	36
Post Lung Transplant (TBD)	18	5	23

Targeted surveys have also been used to obtain broader feedback from the individuals living with the disease. In 2017 and 2018, the CFF conducted surveys of individuals with CF, family members, and health care providers to inform the scope of upcoming guidelines [23-25]. These surveys provided insight that was otherwise unknown to the clinical committee members and informed the scope of the guidelines.

In 2017, the CFF piloted a focus group engagement strategy for the development of one guideline. This pilot included spouses and individuals with CF in a focus group lead by the psychiatrist and the adult with CF on the guideline committee. The focus group was able to provide insight from their experience to help to fill a gap in the CF-specific literature [26]. This supplemental group allowed the committee to hear from multiple individuals with CF and spouses of adults with CF, ensuring that broader perspectives and experiences informed guideline development. The input of the patient and family members on this focus group informed the guideline committee of previously unknown psychosocial barriers that contributed to suboptimal outcomes.

### Communication

Since 2014, the CFF has developed public-facing material to support its guidelines. Previously, materials assisting clinicians in the implementation of these guidelines were developed and shared by the members of the guideline committee via listservs with other clinicians. Individuals with CF/family members on the committee are asked to provide feedback on the resources created to ensure a wider understanding of the guidelines, or other patients and family members are recruited through Community Voice. These resources are disseminated through emails to CF Care Center staff, CFF email listservs, Community Voice, and are posted to the CF Foundation website [27].

### Overview

**Figure 1:**
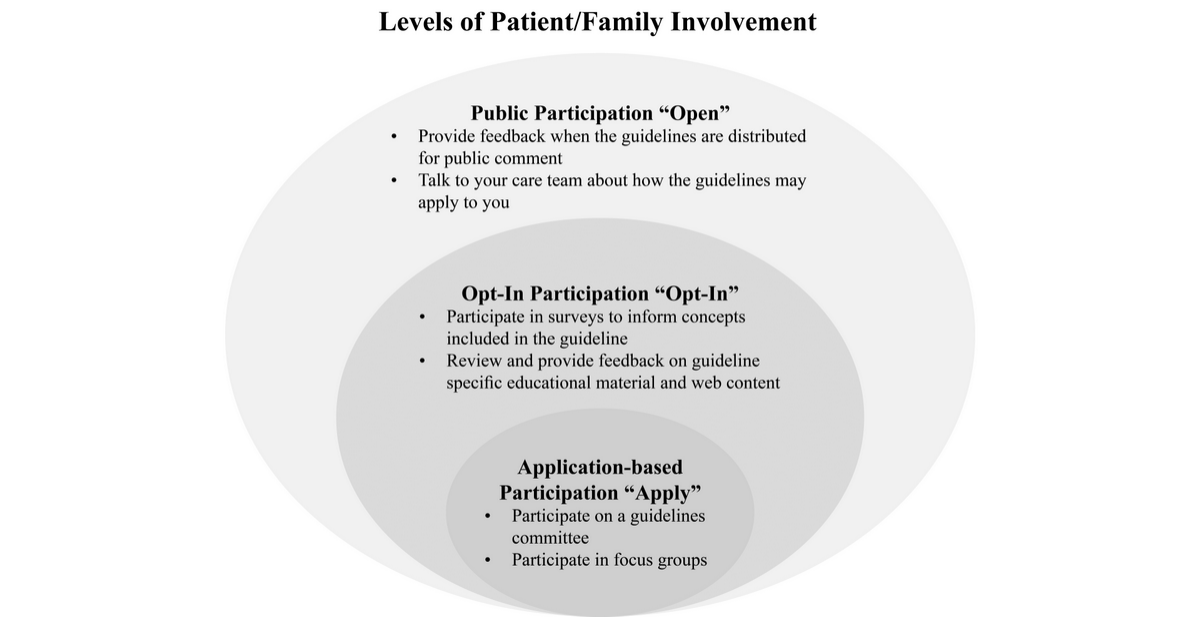
Opportunities for PPI in CFF Guidelines and Guideline Implementation: There are three levels at which individuals with CF and family members can participate in guideline development and implementation. 1. Anyone: these opportunities are open to all patients and families. 2. Opt In: These opportunities require patients and families to sign up for Community Voice to learn about these methods of influencing the guidelines and guideline implementation. 3. Apply: These opportunities require individuals who have opted-in to Community Voice to apply to participate in these projects.

Guideline development via a partnership with clinicians, individuals with CF, and families enables productive interactions between care teams and patients. The concepts laid out by the GIN toolkit, participation, consultation, and communication, provide substantial opportunities for patients, families, and care providers to implement the chronic care model [28] and improve health outcomes. While CF care is provided within the framework of the chronic care model [3], the concepts presented here can be adapted to other patient groups. Before the CFF had access to the active patient and family population through Community Voice, many of the patients and families included in CFF guideline development were recruited by clinicians serving on the guideline committees. An overview of opportunities for patients and families to participate in CFF guideline development and implementation are outline is provided in Figure 1.

### Challenges

Current CFF guideline committees are encouraged to include two individuals with CF on each committee, in addition to at least one family member, ensuring the inclusion of more than one individual’s perspective and preventing the members with CF from feeling that they must represent the entire patient population. It enables the voice of an individual with CF to be present even if the other individual with CF becomes too sick to participate. However, including multiple individuals with CF has unique challenges. The CFF Infection Protection and Control guidelines, and CFF policy, recommend that only one person with CF attends any CFF-sponsored indoor event to decrease the potential for cross-infection [29]. The CFF uses virtual meeting platforms to enable the participation of more than one individual with CF.

The CFF’s relationship with individuals with CF and their family members has been increasingly cultivated with the development of Community Voice. Individuals with CF and family members are now able to apply to participate in CFF projects like guideline committees. By 2019, it had over 1150 members, with participants from all 50 states. While there is broad representation in Community Voice, it does not reflect the entire CF population, as people must sign up to become a member. Members choose what types of projects they would like to hear about and participate in based on their interest and level of time commitment [30]. “Opt-in” membership, and recruitment options, can place limits on the variety of patient and family experiences and perspectives participating in guideline development and implementation.

Proactive partnerships between clinicians, patients, and families may lead to more actionable recommendations at the point of care. However, research is needed to fully understand the influence of PPI on the actionability of guidelines. Research on the effect of including patients and family members on the review of educational materials is needed. Understanding how PPI influences these factors is necessary to improve the guideline and related materials development processes.

### Impact

While systemic reviews exist on the impact of patients in the setting of advisory councils [31], there is limited systematic evidence of the impact of the presence of an individual or family members on the formation of guidelines. This report has highlighted many areas where the input of individuals with CF or their family caregivers improve the focus and patient-centeredness of CFF guidelines. A critical factor in the guideline development process is the interchange between researchers and clinicians with individuals with CF and family members in real-time as PICO questions are created, the evidence is evaluated, and the outcomes are determined. The presence of an individual or family member living with the disease transforms the process from an academic exercise into a meaningful exploration of those questions and outcomes that have an impact on their daily life. These discussions also branch into areas that might not have been considered without the presence of these individuals.

Despite the limited published evidence of the impact of PPI on guideline development, the authors believe that including patients and families in guideline development has improved the guideline development process. In order to better assess the impact of this process of inclusion, in July 2019, the CFF surveyed clinical committee members who participated in a recent guideline committee that included at least one individual with CF or a family member of a person with CF. Fifty-seven individual responses were obtained from the 176 non-patient/family guidelines committee members (32% response rate). Ninety-three percent of respondents agreed or strongly agreed with the statement that the presence of a person with CF or a family member of a person with CF improved the guidelines formation process. Sixty-three percent of respondents agreed or strongly agreed that the involvement of these individuals improved the PICO questions chosen, and 89% agreed or strongly agreed that the presence of these individuals improved the selection process for outcomes considered to be important. The survey also asked whether the presence of individuals with CF and their family members would constrain discussion. Only 9% of respondents agreed or strongly agreed that this was an issue.

An open-ended question in the survey asked the clinical guidelines committee members to describe what they found helpful about the inclusion of a person with CF and/or a family member of a person with CF on the committee. Of the 57 responses, 56 were uniformly supportive of the role. The responses were typified by one committee member who stated, “I learned so much from their presence. I valued their involvement very highly. I was able to ask them questions that I never really thought to ask patients before, and the experience was incredibly informative. Also, it helped shape my ideas of what questions we should be asking and how we should be tailoring care in consideration of how patients are directly impacted.” Another prevalent observation is summed up by another participant: “This was my first experience having a patient representative to help guide professional questions and decision-making. It was extremely valuable and provided a real-world representation of the needs in patients with CF who suffer with chronic medical issues. It was also enlightening to have the adult with CF indicate how her drug-induced hearing loss has affected her life and how she wasn’t provided much information or guidance about this risk during her treatments. I think having both a parent representative and an adult patient with CF was critical to keep the focus on ‘patient needs’ rather than ‘clinician wants’ during our discussions.” According to this participant, the patient representative on this particular guideline committee helped guide the PICO question development process by sharing a personal experience and how it impacted her CF care.

While some data can be found in the literature about the clinical perspective on patient and public involvement, information on the patient and public experience is lacking [32]. In October 2019, a similar survey was distributed to 26 patients and family members who participated in recent guideline development (the 2014 Infection Prevention and Control committee to present committees). Eleven of the 26 patients and family members who were contacted responded to this survey (42% response rate). Eighty-two percent rated their overall experience on the guideline committee(s) as above average to excellent, with only one rating their experience as average and one rating their experience as very poor. Ninety-one percent of the respondents somewhat agreed or strongly agreed that the presence of someone with CF/caregiver/significant other improved the guidelines formation process, and all somewhat or strongly disagreed that the presence of someone with CF/caregiver/significant other inhibited discussion. Seventy-two percent somewhat or strongly agreed that the presence of someone with CF/caregiver/significant other improved the PICO questions chosen. Ninety-one percent somewhat or strongly agreed that the presence of someone with CF/caregiver/significant other improved the outcomes considered to be important.

When asked if they felt that the guideline benefited from their inclusion on the committee as a person with CF/caregiver/significant other, 10 of the 11 respondents felt the guideline benefited from their inclusion. The responses can be exemplified by one individual who stated, “[E]veryone brought a different view to the discussion and mine was not medical but that of a parent which very much plays a role in the care of the patient.” Another indicated, “I believe I was able to articulate unmet needs in current [CF] care that the … guideline could address.”

The individuals with CF and family members included on guidelines committees also keep the committee focused on the variation in experiences within the CF community. These members often remind the committee of the various choices in care and patient priorities, especially around advanced stages within the disease or during transplantation decisions. Their presence has ensured that the committee remembers the variety of care pathways available to targeted patients in the population and that these choices are considered when developing the PICO questions and recommendations. Textbox 1 presents a patient’s perspective on the guideline development process.

**A patient’s perspective.**

The best medical care is a partnership between patients, families, and clinicians. As a patient, there are a few important ways we contribute to the guideline development process. While CF clinicians are no doubt experts in cystic fibrosis, we are the experts on where the “rubber meets the road” in CF care. Having a voice and a vote ensures that guidelines are feasible and more likely to be accepted by the wider CF community. When not directly participating on a specific committee, the opportunity to comment on guidelines is important as it still gives me a voice and input on the guidelines that will affect my care. Our experiences as patients or family members of someone with CF give us a unique viewpoint that often brings up symptoms or issues that might go unrecognized, and therefore left out of guidelines. Finally, there is the feeling of empowerment that comes with being treated as a colleague and not just a patient [33]. The role of guidelines continues to grow as evidence-based medicine becomes the standard. Those guidelines inform the care we receive and are expected to adhere to. Coproduction of guidelines is vital to ensuring that patient wishes and needs are always at the center of guideline development.

## Conclusion

The inclusion of PPI in CFF guideline development since 2004 has dramatically strengthened the culture of the organization’s guideline development. With the advent of Community Voice, the CFF has been able to reach and partner with a broader range of individuals with CF and their family members, allowing more perspectives to be heard. Despite disease-specific challenges, the CFF has been able to utilize technology to incorporate multiple patient and family perspectives into guideline development. These voices also improve the way the CFF talks about and develops educational material related to the guidelines. Reviews of these materials by individuals with CF and family members ensure that the language used is understandable and culturally appropriate.

The surveys that were conducted show that clinicians, patients, and family members believe that the lived experience is an essential aspect in the creation of guidelines. This feedback has encouraged CFF to continue to explore additional ways to involve community members in guideline development and implementation.

The methods CFF uses to include patients and families in guideline development can be adapted to other conditions, both chronic and acute, as all patients and their family members can provide insight into their experiences with their conditions. While CFF has a highly connected and activated population mobilized through Community Voice, partnerships between clinicians, patients, and family members can be built and fostered in many different ways, including one-one relationships, quality improvement projects at the local level, and developing overarching care guidelines for an entire disease population.

Overall, the importance of patient and public involvement and partnership in CFF guideline development was summarized by one individual with CF/family member who stated in the survey that “[t]he personal insight on how things truly are from a CF patient or caregiver is invaluable.”

## Abbreviations

CF: cystic fibrosis
CFF: Cystic Fibrosis Foundation
IOM: Institute of Medicine
GIN: Guidelines International Network
PPI: patient and public involvement
